# Enhancement of cartilage repair through the addition of growth plate chondrocytes in an immature skeleton animal model

**DOI:** 10.1186/s13018-019-1302-y

**Published:** 2019-08-15

**Authors:** Ryszard Tomaszewski, Łukasz Wiktor, Artur Gap

**Affiliations:** 1 Department of Pediatric Traumatology and Orthopedy, Silesian Medical University, Katowice, Poland; 2 Institute of Physics, University of Silesia, Katowice, Poland

**Keywords:** Articular cartilage, Chondrocyte, Trauma, Histological scoring system

## Abstract

**Background:**

The treatment of articular cartilage damage is a major clinical problem. More often, this clinical issue affects children, which forces doctors to find the best treatment method.

**Methods:**

The aim of this experimental study on 2-month-old Landrace pigs was to compare the results of two cartilage defect treatments: (1) filling the cartilage defect with a scaffold incubated with bone marrow aspirate supplemented with growth plate chondrocytes (the CELLS group) and (2) filling the cartilage defect with an empty scaffold implanted after drilling the subchondral bone (the CTRL group). The treatment outcomes were assessed macroscopically and microscopically.

**Results:**

Based on the macroscopic evaluation, all animals showed a nearly normal morphology, with an average of 9.66/12 points (CTRL) and 10.44/12 points (CELLS). Based on the microscopic evaluation, 1 very good result and 8 good results were obtained in the CTRL group, with an average of 70.44%, while 5 very good results and 4 good results were obtained in the CELLS group, with an average of 79.61%.

**Conclusions:**

(1) Growth plate chondrocytes have high chondrogenic potential and thus offer new possibilities for cartilage cell therapy. (2) The implantation of a scaffold loaded with bone marrow-derived MSCs (mesenchymal stem cells) and growth plate chondrocytes into a cartilage defect is a good therapeutic method in immature patients. (3) Cartilage repair based on a scaffold with bone marrow aspirate-derived cells supplemented with autologous growth plate chondrocytes achieves better results than repair with marrow stimulation and a hyaluronic acid-based *scaffold* (overall microscopic rating). (4) Chondrocyte clustering is a manifestation of the cartilage repair process but requires further observation.

**Electronic supplementary material:**

The online version of this article (10.1186/s13018-019-1302-y) contains supplementary material, which is available to authorized users.

## Background

Due to its specific structure and highly specialised function, the treatment of damage to articular cartilage is a major clinical problem. Moreover, its structure, which consists of chondrocytes surrounded by an extracellular matrix, and its avascular character result in a low regenerative capacity. The chondron framework and orientation are closely associated with the microarchitecture, function, and location of articular cartilage, which further complicates the regeneration process. A number of different treatment methods have been developed for both early and advanced stages of cartilage damage, but a definitive method has not yet been determined. Among all of the surgical procedures used for the treatment of cartilage damage, the following should be distinguished: arthroscopic debridement, bone marrow stimulation, osteochondral autografting, autologous chondrocyte implantation (ACI), and matrix-induced autologous chondrocyte implantation (MACI) [1, 2]. The continuous development of regenerative medicine creates new possibilities for cartilage defect treatments involving cell therapy. Isolated and specially cultured cells can be injected into the joint, or via tissue engineering techniques, and can be implanted on special substrates directly in the site of cartilage damage. A variety of cells have been used, as reported in the literature, including autogenic chondrocytes, allogenic chondrocytes, mesenchymal stem cells (MSCs; most widely used; can be derived from bone marrow, blood, synovium, and synovial fluid), and human pluripotent stem cells (embryonic stem cells, ESCs; induced pluripotent stem cells, iPSCs); however, the best cells have yet to be identified [3–12]. The aim of our study was to compare the effects of treating cartilage damage with the two currently most popular techniques: marrow stimulation and autologous cell therapy. We chose the marrow stimulation technique as a reference point because it is a one-step method that does not require expensive instrumentation and has been widely used for many years. Based on the current literature, cell therapies are rapidly developing, with promising results [3]. Chondrocytes and MSCs are the most commonly used cells in cartilage tissue engineering [2, 4]. In our opinion, the key point is to find a simple and optimal one-step surgical cartilage repair technique that restores hyaline cartilage. Due to the aforementioned facts, our study was designed to evaluate the effects of biological therapy based on the use of bone marrow-derived MSCs along with young chondrocytes isolated from growth plates. The proposed method combines the MACI technique with the clinical application of MSCs.

Our research was carried out using a developmental animal model because in everyday practice, we more often diagnose articular cartilage injuries in children in the Orthopedics Department. We wanted to focus on immature animals to mimic cartilage repair in children. Cartilage regeneration potential in children has been described to be more potent compared with adult patients. Moreover, children and adolescents have a greater capacity to spontaneous cartilage healing which correspond with better outcome, both after conservative and surgical treatment. Due to abovementioned, problem of cartilage damage in children should always be considered individually.

## Materials and methods

The study group consisted of 10 pigs at the age of 12 weeks. Each animal was assigned a number, and the knee joints were divided into right (R) and left (L). Both hind legs of all animals were operated on; one side was treated with a hyaluronic acid-based *scaffold* and bone marrow cells via the marrow stimulation technique (the CTRL group), and the other side was treated with bone marrow cells from marrow aspirates supplemented with immature chondrocytes isolated from growth plates (the CELLS group). In the first stage of the experiment, in the operating room with the animal under analgosedation (atropine sulphate 0.05 mg/kg s.c., Polfarmex, Kutno; ketamine hydrochloride 3 mg/kg i.m., Biowet, Puławy; and xylazine hydrochloride 1 mg/kg i.m., Riemser), we performed bone marrow aspiration.

### Bone marrow harvesting

We used the Mini Marrowstim™ Concentration System (BIOMET). Each time, a bone marrow biopsy was performed from the posterior iliac spine to aspirate 30 ml of specimen (3 ml of heparin with 27 ml of bone marrow aspirate). The specimens were centrifuged for 15 min at 3200 rpm, yielding a 3-layer distribution consisting of a plasma-rich layer, a cell-rich layer, and a red blood cell layer.

### Cell-rich concentrate preparation

The isolated cell-rich concentrate was rinsed with Ham’s F12. The gained solution was then centrifuged twice at 200 x g for 5 min and rinsed each time with Ham’s F12.

### Growth plate chondrocyte isolation

Using the trepanning needle, which we used to aspirate the bone marrow, we retrieved a biopsy of the iliac growth cartilage, from which we isolated juvenile chondrocytes according to the following scheme: the growth plate sample was cut into 1 mm^3^ fragments and then washed with Ham’s F12 (PAN Biotech, P-04-14500). The preparations were digested with Collagenase NB 4 (SERVA, 17454.02) at a concentration of 0.3 U/ml in Ham’s F12 at 37 °C for 6 h. The solution was then centrifuged twice at 180×*g* for 10 min and rinsed each time with Ham’s F12. The obtained cells were suspended in 100 μl of Ham’s F12 with 20% serum. For each preparation, we controlled the process of chondrocyte isolation from the growth plate by microscopy.

### Scaffold preparation

We cut the Hyalofast® scaffold into 20 equal fragments similar to the predicted size of the joint cartilage defect. We chose Hyalofast because in the degradation process, hyaluronic acid is released into the lesion, creating a microenvironment rich in HA. Moreover, MSCs combined with Hyalofast have been shown to differentiate into chondrocytes for hyaline-like cartilage regeneration [13, 14]. We divided the scaffolds into two groups. In the first group, after rinsing with Ham’s F12, the scaffolds were incubated with cell-rich concentrate for 5 h and then with chondrocytes isolated from the iliac growth plate for another 7 h. In the second group, the scaffolds consisted only of scaffolds without added cells. Thus, the prepared scaffolds were ready for implantation. In stage II of the experiment in the operating theatre, after prior medication according to the scheme shown above, the pigs were anaesthetized using 1% propofol 1.5–2.5 mg/kg i.v. (Fresenius Kabi, Austria GmbH) and fentanyl 2.5 mg/kg m.c. i.v. and inhaled isoflurane 0.5% volume.

### Cartilage damage induction and scaffold implantation

While monitoring the vital parameters of the animals, we performed a mini-arthrotomy separately for the right and left knees. Each time, we made a cartilage defect on the surface of the medial femoral condyle with a diameter of 6 mm. We paid much attention to gentle and thorough removal of the joint cartilage such that the cavity reached the subchondral bone without secondary damage. In one study group (CTRL), we implanted an empty scaffold into the defect and to provide additional cells for cartilage healing, and we perforated the subchondral bone three times for each defect using a 1-mm-diameter Kirschner wire to a depth of 6 mm. In the second study group (CELLS), we implanted a scaffold incubated with cells without drilling the subchondral bone. The experimental scheme is presented in Tables 1 and 2. Every graft was checked prior to implantation to confirm the graft and animal number. Every graft was stabilised with 2 vicryl 5–0 sutures (Johnson & Johnson). After stable graft placement confirmation, we closed the surgical wound and secured the operated limb with a sterile dressing. The animals were allowed to walk freely in their cages.

**Table 1:**
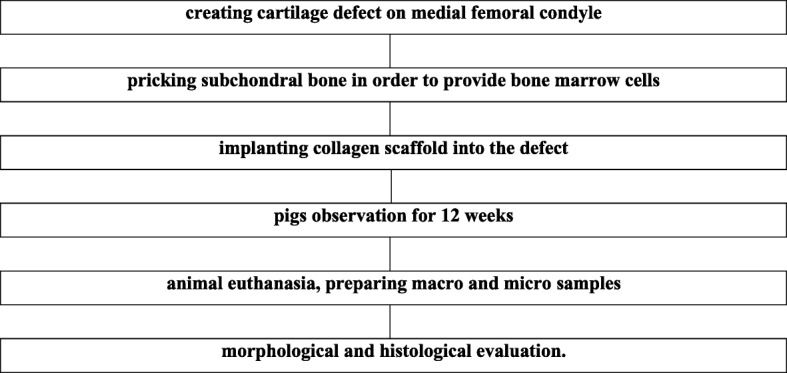
CTRL group–experiment scheme

**Table 2:**
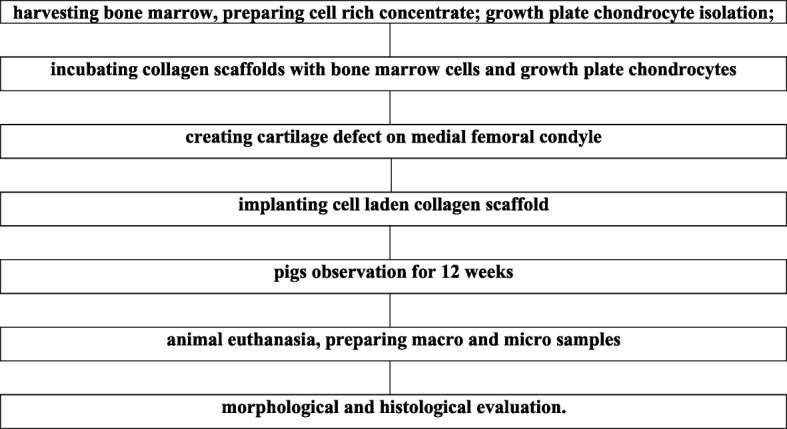
CELLS group–experiment scheme

### Morphological and histological evaluations

After 12 weeks from the day of surgery, the animals were sacrificed (Morbitol 1.6 mg/kg i.v.), and the 20 treated knees were collected. For each specimen, the patella was removed to provide direct visualisation of the joint, which facilitated morphological evaluation of the treated cartilage according to the ICRS classification system (Additional file 1: Table S1). The preparations were then fixed in 10% formaldehyde. After sample decalcification (using TBD-2) and sectioning the material containing regenerated joint cartilage (5 μm thick), the histological preparations were stained with haematoxylin and eosin, Safranin O, and Masson’s trichrome. The histological results were independently evaluated by two pathologists (RT, ŁW) using an Olympus BX51 microscope (Olympus America, Inc., Melville, NY). The ICRS II scale was used for the microscopic evaluation of regeneration (Additional file 2: Table S2).

### Statistical analysis

To verify the difference between the mean measurements in the two groups, i.e. treatment with scaffolds containing cell-rich concentrate and chondrocytes (the CELLS group) and scaffolds with no cells (the CTRL group) for each parameter, we used Student’s *t* test and the non-parametric Wilcoxon test based on Bayesian statistics.

## Results

**Fig. 1:**
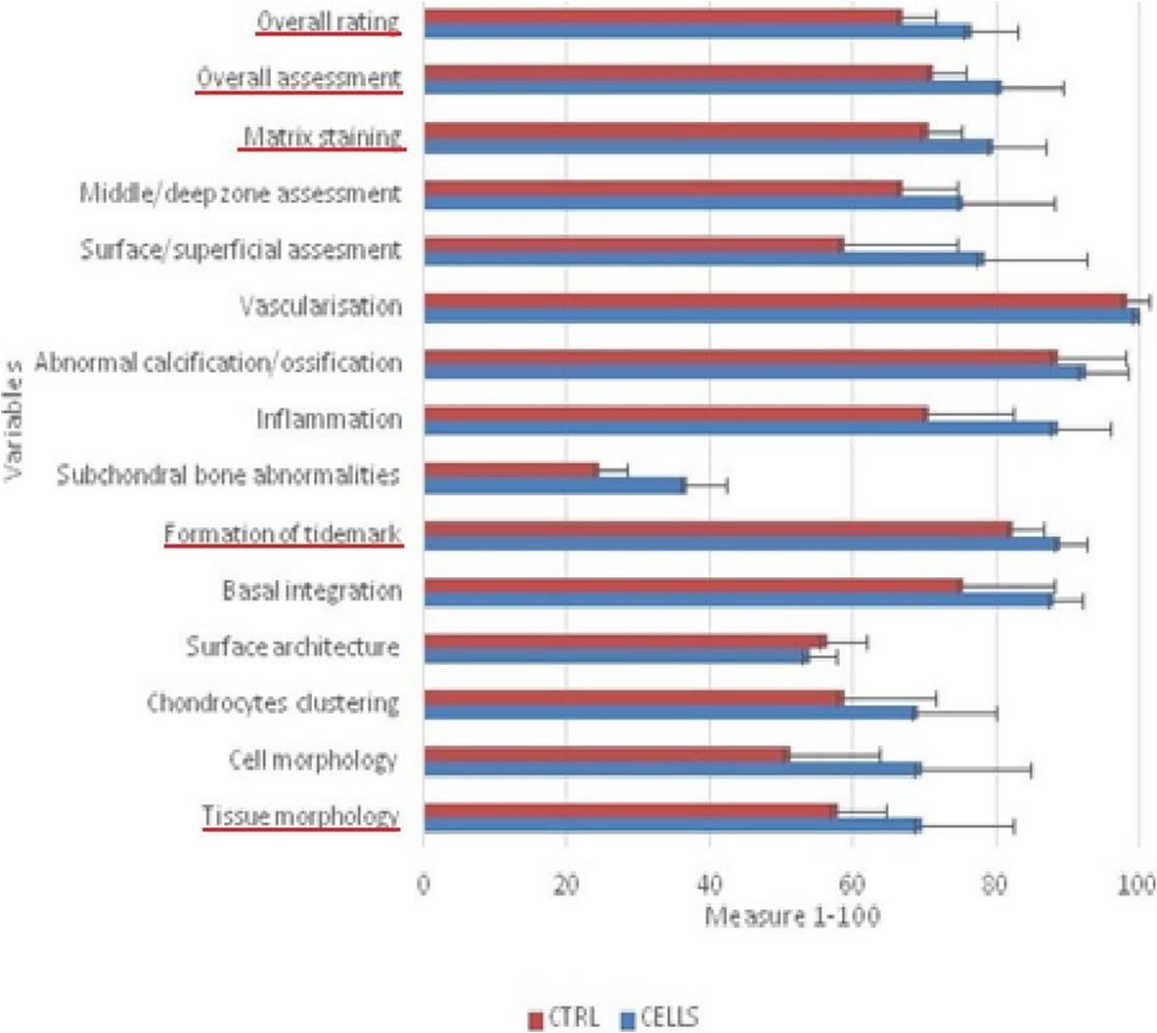
Differences between measurements for group with cells (CELL) and for group without cells (CTRL). Bars represent the mean results. Significantly better parameters highlighted in red

**Fig. 2:**
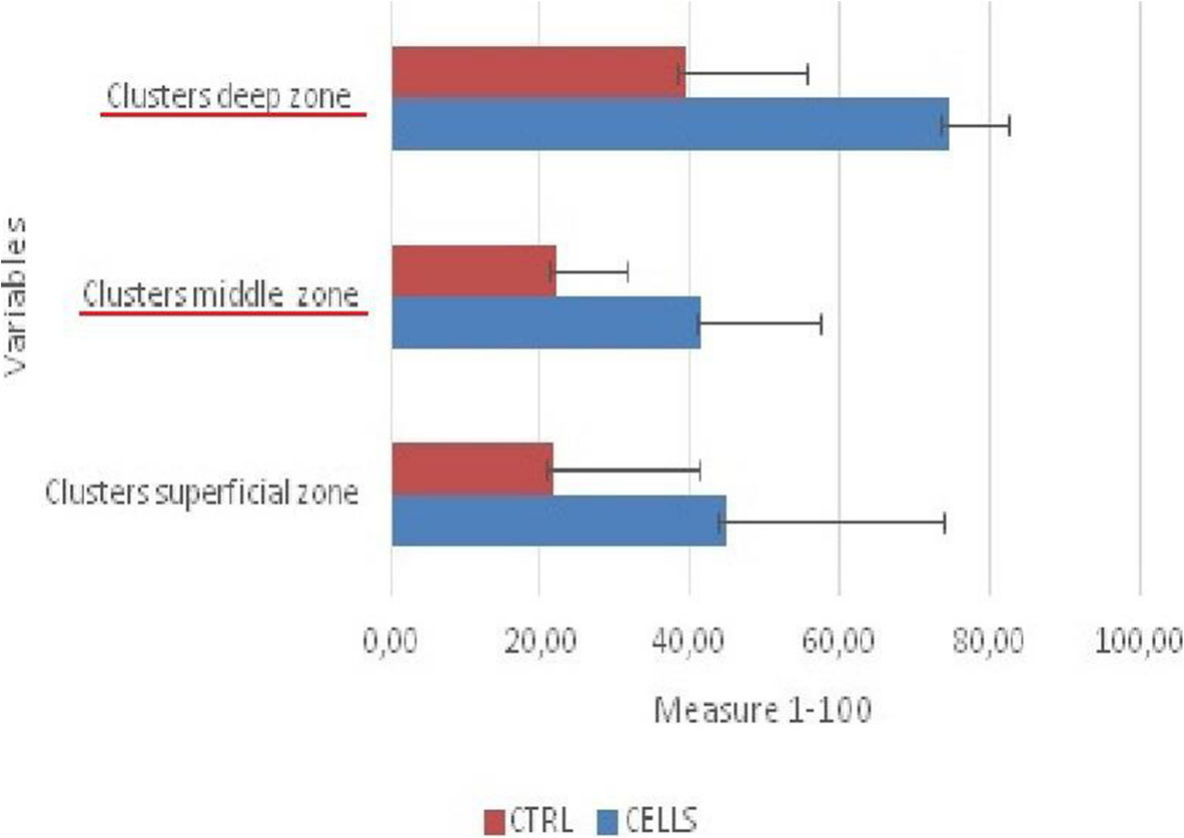
Differences between number of chondrocyte clusters for group with cells (CELL) and for group without cells (CTRL). Bars represent the mean results. Significantly better parameters highlighted in red

**Fig. 3:**
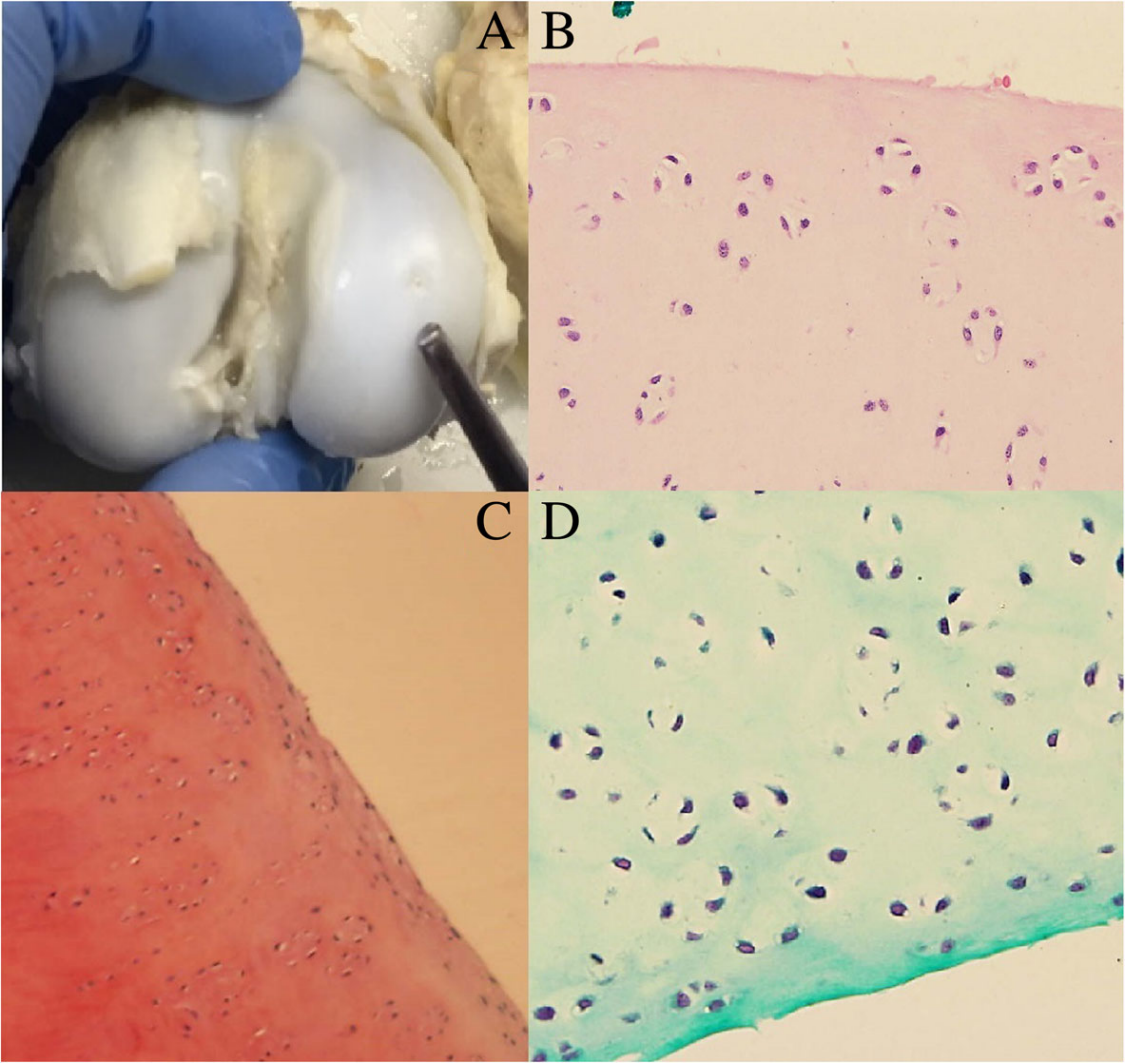
Knee 1P (treated with cells) **a** Macroscopic evaluation. **b** Microscopic view of chondrocyte clusters in regenerated cartilage (200×, H&E staining) **c** Microscopic view of chondrocyte clusters in regenerated cartilage (Safranin O staining). **d** Microscopic view of chondrocyte clusters in regenerated cartilage (Masson staining)

**Fig. 4:**
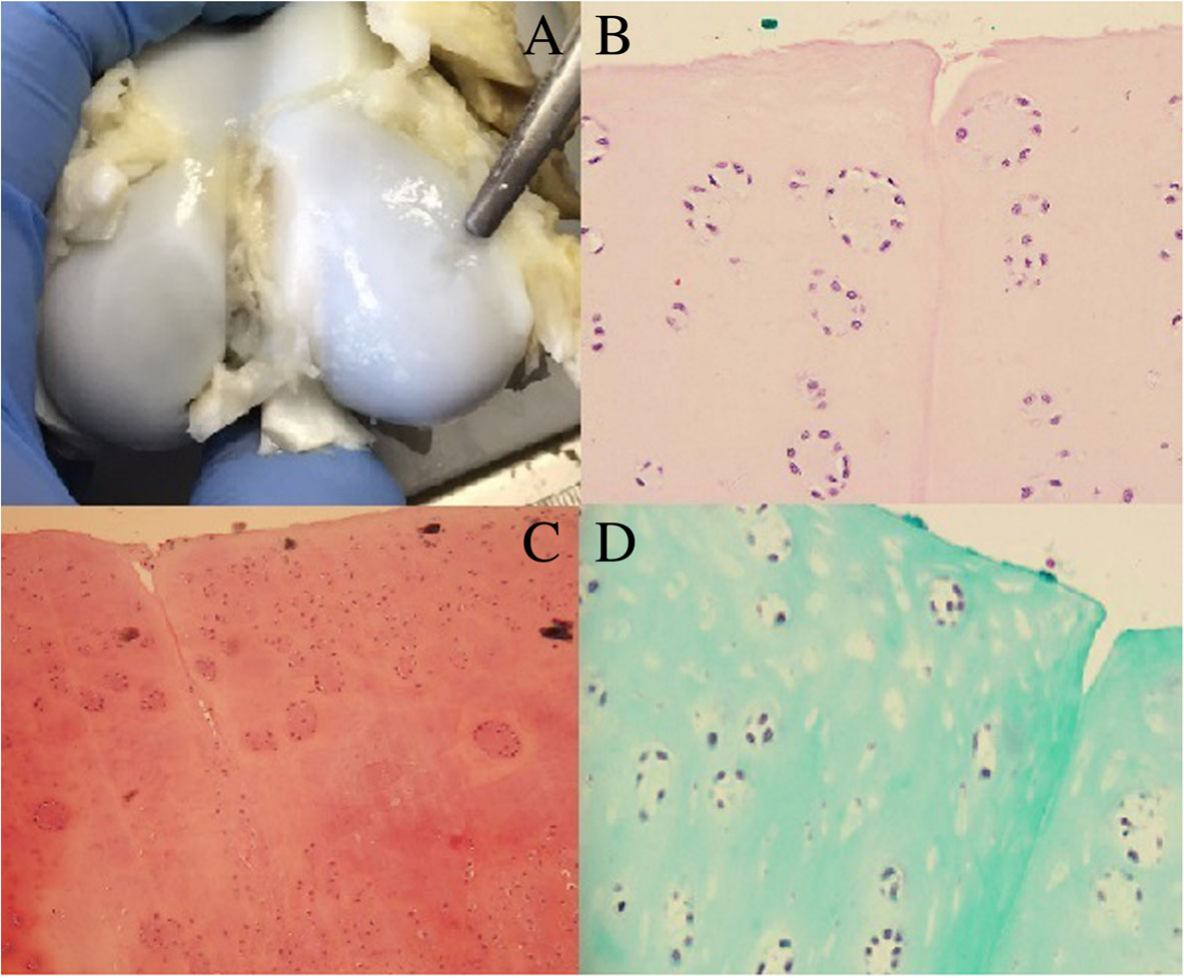
Knee 1L (treated with microfracture technique) **a** Macroscopic evaluation. **b** Microscopic view of chondrocyte clusters in regenerated cartilage (200×, H&E staining). **c** Microscopic view of chondrocyte clusters in regenerated cartilage (Safranin O staining). **d** Microscopic view of chondrocyte clusters in regenerated cartilage (Masson staining)

In one pig (animal designated no. 4), postoperative wound infection was observed. Due to confirmation of the infectious process of the knee (morphologically and histologically), preparations from 4R and 4L were excluded from further evaluations. Based on the obtained results, both methods achieved good and very therapeutic effects. Macroscopic evaluation was performed according to the ICRS classification system, and in the group of joints treated with scaffolds without cells, all specimens showed a nearly normal morphology, with an average score of 9.66/12 points. In the group of joints treated with scaffolds incubated with cells, all joints similarly showed a nearly normal morphology, with an average score of 10.44/12 points. Based on the microscopic evaluation, 1 very good result and 8 good results were obtained in the group of joints treated with scaffolds without cells, with an average of 70.44%; in the group of joints treated with scaffolds incubated with cells, 5 very good results and 4 good results were obtained, with an average of 79.61%. The results were averaged and are presented in Tables 3 and 4. The histological evaluation revealed significantly better results in the CELLS group in terms of the tissue morphology (*p* < 0.01), matrix staining (*p* < 0.01), tidemark formation (*p* < 0.001), and overall assessment (*p* < 0.05). The averaged results of all evaluated parameters were better for the CELLS group, indicating a better overall rating (*p* < 0.01). The results are shown in Table 5 and Fig. 1. More frequently, we found subchondral bone abnormalities in the CTRL group. Notably, we did not observe problems with graft integration in the CELLS group, and basal integration in this group was significantly better than that in the CTRL group (*p* < 0.01). With the exception of one pig, we did not observe lymphocytic infiltration as a response to scaffold implantation. We found a correlation between increased vascularization and a poorer overall rating, but it was not statistically significant. The presence of chondrocyte clusters in the two groups was analysed. By assessing the preparations, we found more chondrocytes forming clusters in the CELLS group (Table 6). Student’s *t* test showed a statistically significant difference in the presence of clusters in the middle (*p* < 0.05) and marginal zones (*p* < 0.01). The Wilcoxon test showed a statistically significant difference in the same parameters identified by Student’s *t* test and for clusters in the superficial zone. According to Cohen’s *d*, the strength of the variable for the middle and marginal zones was large, and the strength for the superficial zone was average. The results are shown in Table 7 and Fig. 2. Representative results are shown in Figs. 3 and 4.

**Table 3:** Macroscopic evaluation of cartilage according to ICRS

	1L	2P	3L	5P	6P	7P	8P	9P	10P	1P	2L	3P	5L	6L	7L	8L	9L	10L
Degree of defect repair	3	3	3	3	3	3	3	3	3	3	4	3	3	3	4	3	3	3
Integration of border zone	4	4	4	3	4	3	4	4	3	4	4	4	4	4	4	4	4	4
Macroscopic appearance	3	3	3	2	2	3	3	4	4	4	4	3	3	2	3	4	4	3
Overall assessment	10	10	10	8	9	9	10	11	10	11	11	11	9	9	11	11	11	10
Scaffold with cells—CELLS										+	+	+	+	+	+	+	+	+
Scaffold without cells—CTRL	+	+	+	+	+	+	+	+	+

**Table 4:** Microscopic evaluation of cartilage according to ICRS

	1L	2P	3L	5P	6P	7P	8P	9P	10P	1P	2L	3P	5L	6L	7L	8L	9L	10L
Tissue morphology	68	65	62	71	50.5	40	52	52	60	88	88	84	82.5	45	39	55	80	65
Matrix staining	79	53	55	75.5	32	59	34	47	25	90	89	90	94	30	59	61	68	45
Cell morphology	80	55	80	70	26.5	75	36	54	52	84	79	86.5	91.5	44	56	61.5	62	57
Chondrocyte clustering	40	48	55	65	56	62	60	55	65	45	50	65	55	52	55	52	55	56
Surface architecture	90	79	78	51.5	79.5	96	79	89	79	96	89	88	81	81	82	90	96	90
Basal integration.	90	88	89	75	77.5	85	82	73	82	95	95	94	85	81	84	92	91	85
Formation of tidemark	25	24	30	36	18	22	21	25	21	35	44	42	44	20	34	30	44	38
Subchondral bone abnormalities	84	55	89	48	47	70	90	65	88	93.5	95	95.5	89	76.5	65	91.5	95	96
Inflammation	100	100	100	62.5	82	100	82	75	97	100	100	100	77	80.5	92	89	95	100
Abnormal calcification/ossification	100	100	100	87	100	100	100	100	100	100	100	100	100	100	100	100	100	100
Vascularization	86	20	95	70	52	50	42	45	70	100	92.5	96	94	50	48	55	95	75
Superficial assessment	81	60	72.5	70	45	72	62	62	80	92	91.5	91.5	91.5	38	67	60	72	75
Mid/deep zone assessment	80	62	75	75.5	61	75	71	66	76	89	92	89	82	75	50	83	84	85
Overall assessment	84	65	75	64	61	71	69	74	71	90	93	91.5	80	68	60	78	80	76
Scaffold with cells—CELLS										**+**	**+**	**+**	**+**	**+**	**+**	**+**	**+**	**+**
Scaffold without cells—CTRL	**+**	**+**	**+**	**+**	**+**	**+**	**+**	**+**	**+**

**Table 5:** Statistical analysis

	*t*	BF_10_	*Z*	Cohen’s *d*	95% CI for Cohen’s *d*		CELLS			CTRL		
					Lower	Upper	M	SD	SE	M	SD	SE
Tissue morphology	2.97**	4.04	− 2.134*	0.99	0.16	1.78	69.56	19.11	6.37	57.78	9.98	3.33
Cell morphology	2.23	2.23	− 1.96	0.74	− 0.02	1.47	69.56	22.82	7.61	51.11	18.82	6.27
Chondrocytes clustering	− 1.00	− 1.00	− 0.91	− 033	− 1.00	0.35	69.11	16.50	5.50	58.67	19.25	6.42
Surface architecture	2.11	2.11	− 1.72	0.70	− 0.05	1.42	53.89	5.40	1.80	56.22	8.18	2.73
Basal integration	3.63**	3.64	− 2.55*	1.21	0.32	2.07	88.11	5.82	1.94	75.56	18.78	6.26
Formation of tidemark	6.29***	6.29	− 2.67***	2.10	0.88	3.28	89.11	5.37	1.79	82.44	6.15	2.05
Subchondral bone abnormalities	3.15*	3.15	− 2.43**	1.05	0.21	1.86	36.78	8.09	2.70	24.67	5.43	1.81
Inflammation	1.33	1,33	− 1.15	0.44	− 0.26	1.12	88.67	10.89	3.63	70.67	17.82	5.94
Abnormal calcification/ossification	1.00	–	− 1.00	–	–	–	92.56	8.95	2.98	88.67	14.01	4.67
Vascularisation	2.28	2.28	− 2.08*	0.76	− 0.01	1.49	100.00	0.00	0.00	98.56	4.33	1.44
Surface/superficial assessment	1.80	1.80	− 1.48	0.60	− 0.13	1.30	78.33	21.71	7.24	58.89	23.46	7.82
Middle/deep zone assessment	1.97	1.97	− 1.72	0.66	− 0.09	1.36	75.56	18.78	6.26	67.11	11.22	3.74
Matrix staining	4.07**	4.07	− 2.38*	1.36	0.41	2.26	79.67	11.05	3.68	70.44	6.89	2.30
Overall assessment	2.59*	2.59	− 1.96*	0.86	0.07	1.62	81.00	12.63	4.21	71.33	6.78	2.26
Overall rating	3.388**	6.606	− 2.310*	1.129	0.259	1.959	76.55	9.83	328	66.91	6.76	2.25

*N* = 9*.* Poor effect, *D* > 0.2; average effect, *D* > 0.5; good effect, *D* > 0.8

*p < 0.05

**p < 0.01

***p < 0.001

**Table 6:** Numbers of clusters in specific cartilage layers

	1P	1L	2P	2L	3P	3L	4L	4P	5P	5L	6L	6P	7L	7P	8L	8P	9P	9L	10L	10P
Clusters—superficial zone	+++ (90)	++ (82)	+ (60)	+++ (90)	+++ (92)	+ (20)	−	−	+ (10)	+++ (89)	+ (10)	+ (10)	+ (10)	− (10)	− (0)	− (0)	+ (5)	+ (20)	− (0)	− (0)
Clusters—middle zone	+ (60)	+ (40)	+ (20)	+ (50)	+++ (85)	+ (40)	−	−	+ (10)	+ (60)	+ (20)	+ (10)	+ (20)	+ (40)	+ (20)	+ (20)	+ (10)	+ (30)	+ (30)	+ (10)
Clusters—deep zone	++ (80)	+ (20)	+ (20)	+++ (90)	++ (80)	+ (35)	−	−	+ (25)	++ (75)	+ (55)	+ (45)	++ (80)	+ (20)	+ (55)	++ (65)	+++ (90)	+ (80)	+ (75)	+ (35)

**Table 7:** Statistical analysis

	*t*	BF_10_	*Z*	Cohen’s *d*	95% CI for Cohen’s *d*		CELLS			CTRL		
					Lower	Upper	M	SD	SE	M	SD	SE
Clusters superficial zone	2.15	1.53	− 2.023*	0.72	− 0.04	1.44	44.56	43.76	14.59	21.89	29.02	9.67
Clusters middle zone	2.704*	2.93	− 2.056*	0.90	0.10	1.67	41.67	23.18	7.73	22.22	13.94	4.65
Clusters deep zone	3.429**	6.94	− 2.201*	1.14	0.27	1.98	74.44	11.84	3.95	39.44	24.04	8.01

*N* = 9. Poor effect, *D >* 0.2; average effect, *D* > 0.5; good effect, *D* > 0.8

*p < 0.05

**p < 0.01

***p < 0.001

## Discussion

Articular cartilage is a highly specialised tissue. Due to poor vascularity, its regenerative abilities are very limited [15]. Many authors have emphasised that despite various treatment methods, it is not possible to restore articular cartilage, only cartilage-like tissue [16–18].

In our experiment, we used 2-month-old Landrace pigs. Due to relatively large animal size, we could perform less invasive and more precise surgical procedures. Additionally, we could obtain growth plate chondrocytes. The similar tissue compliance genotype of this model to those of humans will make it easier to relate the results to the human population and implement the tested method in clinical practice [17]. Children and adolescents have a greater potential to spontaneous cartilage healing which correspond with better outcome. Moreover, stem cells in children are present in greater numbers and are much more able to divide than in adults and that is why biological treatment is a good alternative for immature patients. Indications for surgery are comparable for children and adult patients, but due to regeneration potential, there are no clear guidelines designed for children. Type of surgery should always depend on the size, the location, and the stage of the cartilage defect. Determining study conditions, it is very difficult to choose a relatively large cartilage defect size to sufficiently reduce the possibility of spontaneous regeneration because in skeletally immature animals, spontaneous repair almost always occurs. Based on experiments carried out in mature animals, we chose the cartilage defect size to reduce spontaneous cartilage regeneration, and we gently removed the deep layer of articular cartilage to reveal the subchondral bone [17, 18]. As a reference point, we chose to fill the defect with a hyaluronic acid-based *scaffold* combined with drilling the subchondral bone to provide marrow cells without delivering them from another source (CTRL). This is similar to the microfracture technique, which is another marrow stimulation technique. The microfracture technique is not a perfect technique because it does not restore hyaline cartilage but leads to the production of fibrocartilage [10, 11]. In the CELLS group, cells were obtained from bone marrow aspirates. Thus, bone marrow cells were present in both study groups. Chen et al. [19] showed that the depth of subchondral bone drilling determines the quality of cartilage regeneration, which is why we placed great importance on drilling the bone to a depth of 6 mm in our experiment. We have found many papers confirming the beneficial effects of platelet-rich plasma on the process of cartilage defect healing via the microfracture technique with collagen scaffolds, fibrin glue, or connective tissue membranes [20–22]. There are also many papers confirming the beneficial effect of treatment with mesenchymal stem cells (MSCs) obtained from bone marrow aspirates (BMAs) [23–25], including one-step techniques [26, 27]. Despite these reports, a few prospective studies comparing cell transplantation with the microfracture technique have not shown better results with cell therapy [28–30]. By reviewing the literature, we found reports that the chondrogenic potential of chondrocytes and chondrogenic stem cells declines with age, which suggests the much higher regenerative potential of juvenile chondrocytes [31–33]**.** This finding creates new possibilities in cell therapy. However, there are few reports of the use of these cells in clinical trials. In our experiment, we accurately created the study groups to provide almost identical conditions for comparison of the two therapeutic methods. In contrast to Adkisson et al., as a source of juvenile chondrocytes, we used growth cartilage instead of articular cartilage. Based on our results, we conclude that both the microfracture technique and scaffolds loaded with BMA-derived cells and growth plate chondrocytes achieve good results, with better results for the latter approach (microfracture, average microscopic score—70.44%; scaffolds with cells, average microscopic score—79.61%). What is worth emphasising in terms of cartilage regeneration is that we observed the formation of many chondrocyte colonies or clusters. Regarding the histology of cartilage, chondrocyte clusters are typical in osteoarthritis [15, 16]. Other causes of cluster formation include excessive joint loading, articular cartilage damage, and joint immobilisation [33–37]. After analysing a study reported by Lotz et al. [38], we can explain the presence of these clusters by the initial stage of the regeneration process and the response of the newly created cartilage to mechanical loading. In the literature, there are reports of the presence of chondrocyte clusters in cartilage models of repair procedures. Makino et al. described the presence of clusters in cartilage after treatment with osteochondral allografts [39]. The main feature of clusters related to osteoarthritis versus cartilage regeneration is collagen type II expression with no expression of collagen type I or X [40], but we did not analyse this difference in our work. In analogy to the results of a rabbit model [39], in our work, we observed an increased number of clusters in the middle and deep cartilage layers, which, in our opinion, is a manifestation of the cartilage repair process starting from the deeper layers of the cartilage on the subchondral bone side. Undoubtedly, the presence of chondrocyte clusters is an interesting and not fully explained manifestation of the cartilage response to the stress factor and requires further observation. Based on our results, we believe that the addition of immature chondrocytes provides new possibilities for articular cartilage treatment.

## Conclusions

1. Growth plate chondrocytes have a high chondrogenic potential and could thus create new possibilities for cartilage cell therapy.
2. The implantation of a hyaluronic acid scaffold loaded with bone marrow-derived MSCs and chondrocytes isolated from growth plates is a good method for the treatment of cartilage defects in immature patients.
3. Cartilage repair based on the implantation of a scaffold loaded with BMA-derived cells supplemented with autologous growth plate chondrocytes achieves better results than the microfracture technique (overall microscopic rating).
4. Chondrocyte clustering is a manifestation of the cartilage repair process but requires further observation.

## Study limitations

1. The study group was relatively small (ten pigs).
2. Due to the small study group, there was no group consisting of pigs with an untreated cartilage lesion to assess potential for spontaneous regeneration.

## Additional files

Additional file 1: **Table S1.** Cartilage repair assessment ICRS. (DOCX 18 kb)

Additional file 2: **Table S2.** ICRS II. (DOCX 19 kb)

## Data Availability

All research data are available from the Medical University of Silesia, Department of Pediatric Traumatology and Orthopedy, Katowice, Poland.

## Abbreviations

ACI: autologous chondrocyte implantation
BMA: bone marrow aspirate
ESCs: embryonic stem cells
iPSCs: induced pluripotent stem cells
MACI: matrix-induced autologous chondrocyte implantation
MSCs: mesenchymal stem cells
