# Supplementary material for: Enhancement of cartilage repair through the addition of growth plate chondrocytes in an immature skeleton animal model
Source: J Orthop Surg Res. 2019 Aug 15;14:260. doi: 10.1186/s13018-019-1302-y (PMC6694631; doi:10.1186/s13018-019-1302-y)
Supplement: Supplementary file 1 — Table S1. Cartilage repair assessment ICRS. (DOCX 18 kb) [file 13018_2019_1302_MOESM1_ESM.docx]

**Additional file 1: Table S1. Cartilage repair assessment ICRS.**

|  | **Points** |
| --- | --- |
| **Degree of defect repair** | |
| In level with surrounding cartilage | 4 |
| 75% repair of defect depth | 3 |
| 50% repair of defect depth | 2 |
| 25% repair of defect depth | 1 |
| 0% repair of defect depth | 0 |
| **Integration to border zone** | |
| Complete integration with surrounding cartilage | 4 |
| Demarcating border<1 mm | 3 |
| 3/4th of graft integrated, 1/4th with a notable border>1 mm width | 2 |
| 1/2 of graft integrated with surrounding cartilage, 1/2 with a notable border>1 mm | 1 |
| From no contact to 1/4th of graft integrated with surrounding cartilage | 0 |
| **Macroscopic appearance** | |
| Intact smooth surface | 4 |
| Fibrillated surface | 3 |
| Small, scattered fissures or cracs | 2 |
| Several, small or few but large fissures | 1 |
| Total degeneration of grafted area | 0 |

Overall repair assessment:

Grade I - normal: 12 pkt;

Grade II - nearly normal: 11-8 pkt;

Grade III - abnormal: 7-4 pkt;

Grade IV - severely abnormal: 3-1 pkt.
