# Supplementary material for: Enhancement of cartilage repair through the addition of growth plate chondrocytes in an immature skeleton animal model
Source: J Orthop Surg Res. 2019 Aug 15;14:260. doi: 10.1186/s13018-019-1302-y (PMC6694631; doi:10.1186/s13018-019-1302-y)
Supplement: Supplementary file 2 — Table S2. ICRS II. (DOCX 19 kb) [file 13018_2019_1302_MOESM2_ESM.docx]

**Additional file 2: Table S2. ICRS II.**

|  | **ICRS II** |  |
| --- | --- | --- |
| 1. | Tissue morphology (viewed under polarized light) | 0%: Full-thickness collagen fibers  100%: Normal cartilage birefringence |
| 2. | Matrix staining (metachromasia) | 0%: No staining  100%: Full metachromasia |
| 3. | Cell morphology | 0%: No round/oval cells  100%: Mostly round/oval cells |
| 4. | Chondrocyte clustering (4 or more grouped cells) | 0%: Present  100%: Absent |
| 5. | Surface architecture | 0%: Delamination, or major irregularity  100%: Smooth surface |
| 6. | Basal integration | 0%: No integration  100%: Complete integration |
| 7. | Formation of a tidemark | 0%: No calcification front  100%: Tidemark |
| 8. | Subchondral bone abnormalities/marrow fibrosis | 0%: Abnormal  100%: Normal marrow |
| 9. | Inflammation | 0%: Present  100%: Absent |
| 10. | Abnormal calcification/ossification | 0%: Present  100%: Absent |
| 11. | Vascularization (within the repaired tissue) | 0%: Present  100%: Absent |
| 12. | Surface/superficial assessment | 0%: Total loss or complete disruption  100%: Resembles intact articular cartilage |
| 13. | Mid/deep zone assessment | 0%: Fibrous tissue  100%: Normal hyaline cartilage |
| 14. | Overall assessment | 0%: Bad (fibrous tissue)  100%: Good (hyaline cartilage) |
